# State-of-the-Art Smart and Intelligent Nanobiosensors for SARS-CoV-2 Diagnosis

**DOI:** 10.3390/bios12080637

**Published:** 2022-08-13

**Authors:** Sushma Thapa, Kshitij RB Singh, Ranjana Verma, Jay Singh, Ravindra Pratap Singh

**Affiliations:** 1 Department of Chemistry, Institute of Science, Banaras Hindu University, Varanasi 221005, Uttar Pradesh, India; 2 Department of Physics, Institute of Science, Banaras Hindu University, Varanasi 221005, Uttar Pradesh, India; 3 Department of Biotechnology, Faculty of Science, Indira Gandhi National Tribal University, Amarkantak 484887, Madhya Pradesh, India

**Keywords:** nanobiosensors, smart and intelligent nanomaterials, nanomaterials, SARS-CoV-2, COVID-19, biosensors

## Abstract

The novel coronavirus appeared to be a milder infection initially, but the unexpected outbreak of severe acute respiratory syndrome coronavirus 2 (SARS-CoV-2), commonly called COVID-19, was transmitted all over the world in late 2019 and caused a pandemic. Human health has been disastrously affected by SARS-CoV-2, which is still evolving and causing more serious concerns, leading to the innumerable loss of lives. Thus, this review provides an outline of SARS-CoV-2, of the traditional tools to diagnose SARS-CoV-2, and of the role of emerging nanomaterials with unique properties for fabricating biosensor devices to diagnose SARS-CoV-2. Smart and intelligent nanomaterial-enabled biosensors (nanobiosensors) have already proven their utility for the diagnosis of several viral infections, as various detection strategies based on nanobiosensor devices are already present, and several other methods are also being investigated by researchers for the determination of SARS-CoV-2 disease; however, considerably more is undetermined and yet to be explored. Hence, this review highlights the utility of various nanobiosensor devices for SARS-CoV-2 determination. Further, it also emphasizes the future outlook of nanobiosensing technologies for SARS-CoV-2 diagnosis.

## 1. Introduction

**Figure 1:**
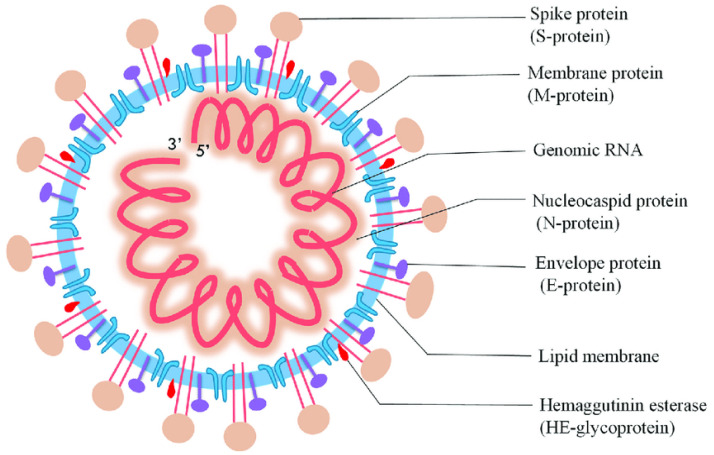
Diagrammatic representation of SARS-CoV-2 (reproduced with permission from Jalandra, R., et al., 2020, 129, 110446 [4]).

In Wuhan, China, on 31 December, an unprecedented respirational disease became apparent and spread rapidly throughout the world. Subsequently, it became evident that this was a new form of coronavirus that had occurred previously, and the World Health Organization (WHO) termed it as the 2019 novel coronavirus (nCoV-2019) [1]. Afterward, the International Committee on Taxonomy of Viruses named it severe acute respiratory syndrome coronavirus 2 (SARS-CoV-2). SARS-CoV-2 belongs to the Coronaviridae and Nidovirales families, which have a single-stranded ribonucleic acid (ssRNA) genome analogous to Middle East respiratory syndrome (MERS-CoV) and SARS-CoV, and alpha (α), beta (β), gamma (γ), and delta (δ) coronavirus are subgroups of this family based on genetic properties. Moreover, SARS-CoV-2 comprises the matrix (M), spike (S), nucleocapsid (N), and envelope (E) structural proteins (Figure 1), and these proteins can be employed as the analyte of interest for the determination of SARS-CoV-2 [2,3].

The devastating pandemic situation has led to approximately 540 million affirmed cases, and beyond 6.33 million fatalities all over the world as of 24 June 2022; therefore, compared to preceding pandemics, SARS-CoV-2 is considerably more infectious, and it shows the utmost contagious efficiency between all the members of the Coronaviridae family [5,6]. Unfortunately, this dreadful condition can continue for an indeterminate duration. Early SARS-CoV-2 symptoms include fever, sore throat, discomfort, loss of taste and/or smell, cough, and respiratory distress. It mainly spreads through the exposure of a normal person to an infected person within the vicinity of 1 m, as while coughing or sneezing, the produced germ particles keep dangling in the atmosphere or may be enclosed in a suspended substance, permitting their transmission through the air [7,8]. Apart from causing health issues, this disease has caused an economic crisis globally. The serious obstacles related to SARS-CoV-2 involve its prevention and prompt diagnosis. Further, presently, no authorized medicines are accessible to combat the SARS-CoV-2 virus [7,9]. Several strategies and investigations have been proposed to prevent further transmission of the SARS-CoV-2 virus, for instance, quarantine, social distancing, the use of face masks, timely detection, and reliable treatment. Many diagnostic methods are accessible for a SARS-CoV-2 assay, and various are under progress [10,11]. Currently, the most popular molecular technique for the ongoing epidemic is reverse transcription–polymerase chain reaction (RT-PCR), which is based on single-stranded nucleic acid amplification tests; this may be performed via brush biopsies or nasal swabs and may give results within 4–6 h. It is a precise method that needs a small amount of RNA. RT-PCR sensitivity and specificity depend on the primer design and assay design; moreover, it is dependent on the proficiency of the individual conducting the analysis as the analysis is automated in most cases. Although these techniques are more remarkable, there are still a few limitations, such as the time taken, the high cost, its unsuitability for rapid detection, and laborious nature [12,13]. Similarly, reverse transcription–loop-mediated isothermal amplification (RT-LAMP) and clustered regularly interspaced short palindromic repeats (CRISPR) testing are used for rapid and point-of-care detection. Moreover, the lateral flow immunoassay (LFA) and enzyme-linked immunosorbent assay (ELISA) have emerged with great potential for point-of-care testing [5,14]. Notwithstanding that the efforts have never come to an end to create quick and exceptional techniques, there is still an absolute necessity for a fast, practical, cheap, and easy-to-use testing approach for diagnosing SARS-CoV-2 quickly and accurately. The most recent development in nanotechnology may provide considerable support for diagnostic and treatment methods in this period of pandemic diseases. Nanotechnology also has an essential role in designing and developing biosensors [15].

Fortunately, biosensors can be an excellent alternative to the traditional tools for quick point-of-care detection, and may be regarded as an analytical tool for determining the SARS-CoV-2 virus. Moreover, a biosensor (analytical tool) is comprised of biological components that can detect the existence of a target ligand and produce a signal which is proportional to the concentration of the analyte. The three main components of the biosensor are the bioreceptor, transducer, and amplifier. The bioreceptor is a biomolecule that catalyzes or binds a specific reaction for a particular analyte to produce a detectable signal, and the transducer converts the recognition event into a measurable signal that can be stored, manipulated, analyzed, and displayed. The amplifier simply amplifies the converted biological signal to an electrical signal [16,17]. The sensitivity and efficiency of the biosensor may be enhanced and developed by using nanomaterials for their fabrication [18]. The nanomaterial size is between 1–100 nm, which is intended to exhibit new characteristics compared to materials without nanoscale features, in particular, better conductivity, and unique thermal, optical, and chemical properties. The application of nanomaterials in the healthcare sector for rapid point-of-care diagnostics, drug delivery, and bio-imaging has resulted in significant interest. Due to their enhanced volume-to-surface ratio and magnetic and electrical properties, the nanostructures of metal and metal oxide (gold, silver, copper, cupric oxide, etc.), carbon materials, quantum dots, and polymers have been used extensively for the fabrication of biosensors [19]. Therefore, the nanomaterial-based biosensor comprises several advantages because of the nanomaterial’s remarkable physicochemical properties, quantum-size effect, and high adsorption. Nanomaterial-based biosensing has been successfully implemented to detect numerous infections, for example, HIV, influenza virus, and hepatitis B virus, so it also has great potential to determine SARS-CoV-2 [2]. This review emphasizes the application and function of various nanomaterial-based biosensing techniques for SARS-CoV-2 because of the urge to develop early diagnostics devices. Consequently, metal-based, carbon-based, quantum dot-based, and polymer-based nanobiosensors have played a crucial part in determining the SARS-CoV-2 virus. Accordingly, this study focuses on nanomaterial-based biosensors, challenges, limitations, and prospects.

## 2. Traditional Techniques and Current Diagnostic Strategies

### 2.1. Traditional Techniques

The ongoing outbreak of the SARS-CoV-2 pandemic threatens worldwide health and has affected every individual’s life in one way or another, which has caused serious concern [20]. Currently, various traditional techniques are employed for screening, detecting, and confirming ongoing pandemic viral infections, for instance, RT-PCR, RT-LAMP, LFA, ELISA, and CRISPR [21,22]. The sensing of viruses involves the act of classifying disease-specific molecules and molecular changes [23,24].

#### 2.1.1. RT-PCR

RT-PCR is a laboratory method typically used to detect the responsible viruses from respiratory secretions. It is a real-time technique frequently used in molecular biology to augment DNA samples. RT-PCR converts the viral RNA to the DNA of SARS-CoV-2 via a reverse transcriptase enzyme. The designing of RT-PCR generally comprises the designing of primers and sequence alignment. The subsequent step includes the testing and optimization of assay conditions such as incubation times, temperature, and reagent conditions [25,26]. The evaluation must aim at the viral genome, for example, the RdRp, N, E, and S genes [27,28,29]. RT-PCR allows early, specific, and sensitive viral detection because it directly detects RNA and enhances the genomic material, although only a low level of the viral RNA is present in the collected biological specimens [30]. However, this technique still suffers from certain practical restrictions. For instance, RT-PCR based on laboratory testing takes around 4–6 h for complete analysis (from sampling to analysis), but when the conveyance of clinical samples to the laboratories is required, diagnosis time often takes 1–2 days, leading to delays in diagnoses; in addition to this, it also requires highly qualified technical personnel and has a high maintenance cost.

#### 2.1.2. RT-LAMP

RT-LAMP, a rapid technology gaining considerable interest in point-of-care (POC) applications, has been applied to the sensing of pathogens such as the SARS-CoV-2 virus. Huang, et al. fabricated RT-LAMP to rapidly test and screen SARS-CoV-2 in 30 min, and in isothermal conditions, the required gene sequence was enhanced [31]. RT-LAMP employs a DNA polymerase and 4–6 primers to bind to six distinct regions on the target genome. There are two inner primers (a reverse and a forward inner primer) and two outer primers in a four-primer system. Compared to RT-PCR, this method is much more straightforward and economical because it does not require an expensive thermal cycler, as the RNA amplification is conducted at a constant temperature. RT-LAMP is cost-effective for mass screening at hospitals, particularly medical centers in rural areas and regional hospitals, and takes 30 min to detect SARS-CoV-2 nucleic acid [23,32]. Despite this, there are some limitations, for instance, cross-contamination due to the formation of aerosols from the RT-LAMP products

#### 2.1.3. ELISA

ELISA is a fundamental method for evaluating immunoglobulin G (IgG), immunoglobulin A (IgA), and immunoglobulin M (IgM), as well as the antibodies of SARS-CoV-2, due to its high sensitivity [33]. ELISA is an analytical technique that can only be performed in laboratories with the support of trained personnel. ELISA is specifically suitable for POC due to its high throughput with variable sensitivity range, its ability to test multiple samples, and its flexibility. Moreover, Liu, et al. reported that ELISA has excellent sensitivity for the acquisition of IgG and IgM antibodies from the nucleocapsid protein (rN) and spike protein (rS) [34].

#### 2.1.4. LFAs

LFAs are qualitative chromatographic assays based on a two-site non-competitive format. In LFAs, the specimen is deposited onto the sample pad of a cassette-like device and moved through a strip via capillary action. LFAs are the POC testing method employed to detect the SARS-CoV-2 antigen or IgM and IgG antibodies because of their minimal cost, rapid time-to-result, simplicity, stability, and effortless production [35,36]. LFA tests can be carried out at home without needing any skilled personnel. Compared to laboratory-based techniques, LFAs are easier, highly portable, and less dependent on trained personnel and equipment [37]. Nevertheless, LFAs have a few limitations, for instance, the target throughput and moderate sensitivity compared to lab-based technologies. Moreover, LFA associated with a weak signal causes reduced sensitivity. The main issue with the LFA technique is that it only works well for symptomatic patients of SARS-CoV-2. Although it works for some asymptomatic patients and gives a true positive result, this is not always a case; sometimes, it gives a false-negative result for asymptomatic patients because it is less sensitive than PCR. Hence, it works better when patients are symptomatic [38].

#### 2.1.5. CRISPR

CRISPR technology currently offers rapid, accurate, in-field, and sensitive detection of SARS-CoV-2, and it also shows extraordinary potential for the detection of nucleic acid. Specific high-sensitivity enzymatic reporter unlocking (SHERLOCK) was fabricated as a sensitive CRISPR-based diagnostic that allows the screening of RNA or DNA [39]. CRISPR-based tests can be employed for POC and can be carried out at laboratories, schools, and airports because the test is easy to carry out and has a simple interpretation of the outcome [40]. Gootenberg, et al. developed a method for detecting nucleic acids, which has been successfully applied to identify human viruses. CRISPR technology provides enormous benefits relative to other traditional nucleic acid detection approaches because it does not require expensive laboratory equipment or skilled personnel [41,42].

### 2.2. Current Diagnostic Techniques

**Figure 2:**
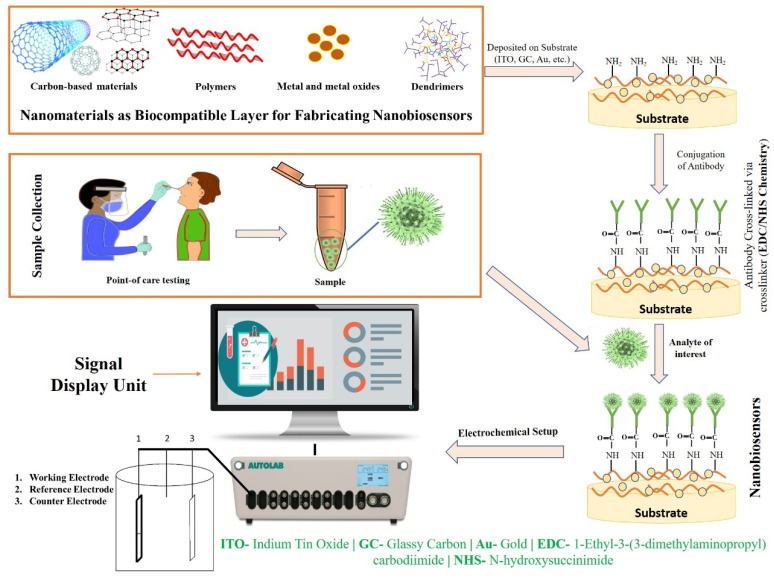
Fabrication strategy and working mechanism of nanobiosensors.

The aforesaid laboratory techniques are currently being used in medical applications for the screening, detection, clinical management, and prohibition of major complications under investigations of SARS-CoV-2. However, these techniques have certain limitations. Therefore, there is a necessity for a productive method for the early detection of viruses, because traditional tools cannot work as a panacea [43,44]. Thus, biosensors seem to be one of the most promising alternatives to traditional diagnostics. In recent years, biosensors have garnered significant interest for screening pathogenic viruses, including SARS-CoV-2, due to their ability to provide sensitive, rapid, low-cost, and portable alternative platforms to traditional diagnostics [44]. Based on the transducer, a biosensor can be electrochemical, piezoelectric, or optical [45]. Most of the designed sensors use an electrochemical- or optical-based detection platform. An electrochemical biosensor recognizes the biological action of analytes and converts these changes into measurable signals in the form of potential, impedance, and current [46]. Electrochemical biosensors can be an effective alternative tool for the screening and detection of the SARS-CoV-2 virus in the clinical diagnosis, and it could also be used in outbreak areas where proficient testing laboratories are hard to reach due to their several advantages, for instance, low-cost, portability, and miniaturization [47,48]. In an optical biosensor, an optical signal is emitted which is directly proportional to the concentration of the analyte. Anfossi, et al. fabricated the dual-functioning optical/chemiluminescence immunosensor to detect SARS-CoV-2-specific IgA in saliva and serum samples. This test effectively detected 25 serum and 9 saliva samples collected from infected and/or recovered persons via colorimetric lateral flow immunoassay (LFIA) [49]. Piezoelectric biosensors, due to a mass bound on the piezoelectric crystal surface, measure the alterations in the resonant frequency. The main challenge for constructing biosensors is to capture a signal of very low magnitude which takes place between the bio-receptor and the analyte, which is improved by employing nanomaterials. Recent studies have reported that nanomaterial-based biosensors (Figure 2) have shown wide application in the development of biosensors and can be a substitute tool that allows for the direct identification of analytes, as they possess high selectivity and cost-effectiveness, are easily fabricated, reliable, sensitive, and user-friendly, and are the best technique for point-of-care test (POCT) diagnostics [44,50,51].

## 3. Nanobiosensors for SARS-CoV-2 Diagnosis

**Figure 3:**
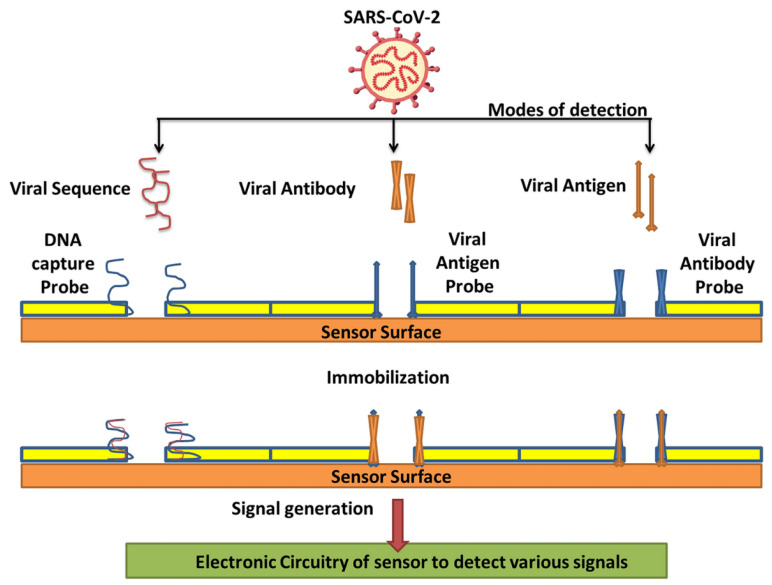
Various modes of detection for SARS-CoV-2 via nanobiosensors (reproduced with permission from V. Chaudhary, et al., Nanotechnol. Environ. Eng. 6 (2021) 8 [7]).

Countless efforts have been made to develop several nanomaterial-based biosensors to identify various diseases such as the hepatitis C virus, porcine epidemic diarrhea, human immune deficiency virus, and SARS-CoV-2 virus in the last several years [2,52]. The nanomaterial-based biosensor has been the focus because of its exclusive detection properties, which allow the sensing of analytes such as aptamers, nucleic acids, and proteins. Nanomaterials, namely carbon-based, polymer-based, quantum-dot, and metal-based nanomaterials, are frequently intended for biosensing applications to diagnose SARS-CoV-2 infection through various modes of detection (Table 1)—such as viral sequence, antibody, or antigen, as illustrated in Figure 3—as they have already exhibited their potential for detecting other viruses. Thus, nanomaterials and their composites have unique properties such as excellent adsorption, quantum-size effects, and great surface-to-volume ratios, which play a vital role in refining the performance of biosensors [53].

### 3.1. Metal and Metal Oxides-Based Nanobiosensors

**Figure 4:**
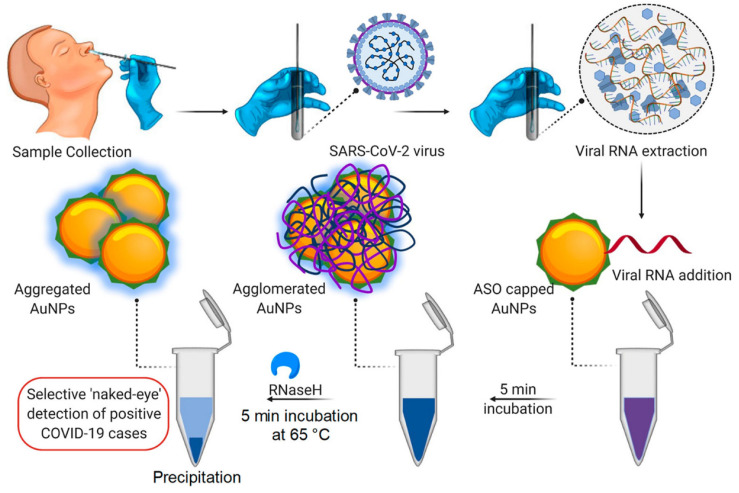
Illustration of Colorimetric assay for SARS-CoV-2 determination through N-gene (reproduced with permission from Moitra, P., et al., ACS Nano, 2020, 14, 7617–7627 [54]).

Metal nanoparticles have created enormous potential for treating and diagnosing pathogenic viruses, as nanoparticles, namely copper (Cu), Gold (Au), iron (Fe), and silver (Ag), display excellent antibacterial and antiviral activity, contrary to other nanoparticle types [7,66]. NP biosensors comprise a biological substrate or immobilized ligand, which undergoes conversion with the occurrence of an analyte. The analyte induces modification in the substrate composition, thus imparting a change in the surroundings of the NPs, resulting in changes in the aggregation status of the NPs and leading to detectable color changes [67]. Gold nanoparticles (AuNPs) have recently garnered consideration by virtue of their unique optical, physical, catalytical, biocompatible, and electrical properties; furthermore, the biosensing of virus infection is mostly performed by gold nanoparticles [68,69]. NPs could conjugate with receptors to form aggregates. AuNPs couple with antibodies specific to the SARS-CoV-2 antigens, and in the presence of antigens, AuNPs aggregate. The aggregation of AuNPs leads to the optical absorption spectrum, which undergoes a red shift (larger wavelength) and the solution changes to a blue spectrum [70]. Therefore, various gold-nanoparticle-based biosensors can determine the SARS-CoV-2 infection via the colorimetric method. For instance, Moitra, et al. conducted a colorimetric bioassay with gold-nanoparticle-capped thiol-modified antisense oligonucleotides (ASOs) to determine SARS-CoV-2 by considering nucleocapsid phosphoprotein (N-Gene) as an analyte of interest. The methodology uses a comprehensive targeting method facilitated by four of the ASO sequences (which are selected based on their relative binding energies with the target sequence and binding disruption energies) covering two regions of the target gene sequence (N-gene) simultaneously. Additionally, with the occurrence of the target RNA sequence of SARS-CoV-2, the thiolated ASO-capped AuNPs agglomerate and a modification in its SPR is demonstrated; this is further augmented with the addition of RNaseH (which cleaves the RNA strand from the RNA–DNA hybrid), leading to the visual naked-eye detection of AuNPs (Figure 4). The identification is made through surface plasmon resonance with a red shift of approx. 40 nm in the spectra of absorbance. Further, this same method was carried out for MERS-CoV viral RNA determination, but no alteration in absorbance was observed [54].

**Figure 5:**
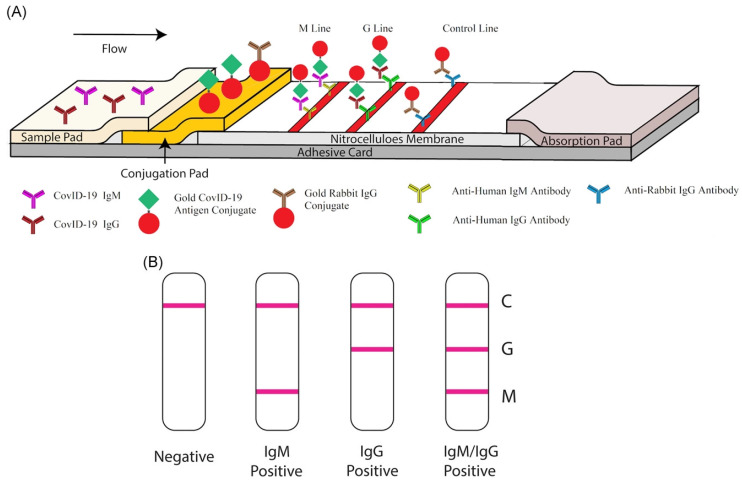
Rapid detection kit for SARS-CoV-2 based on combined IgM-IgG antibody: (**A**) The LFIA device for POC diagnostics of SARS-CoV-2. (**B**) Demonstration of the test results (reproduced with permission from Li, Z., et al., J. Med. Virol., 2020, 92, 1518–1524 [72]).

Additionally, an unusual effort was made with gold nanoparticles to identify the disease and bacteria in laboratory and medical samples. Gasbarri, et al. illustrated the antiviral activity of the sulfonated compound against SARS-CoV-2 by using gold nanoparticles coated with decanesulfonic acid in the micromolar and nanomolar range. Previously, sulfonated compounds were shown to be active against the heparan sulfates (HS) virus [71]. Further, Li, et al. fabricated a gold-nanoparticle-based LFIA strip that can concurrently diagnose IgM and IgG antibodies for SARS-CoV-2, which might be employed to quickly screen asymptomatic and symptomatic patients of SARS-CoV-2 in laboratories and hospitals in real-time, POC diagnostics (Figure 5A). Additionally, a combined IgG-IgM antibody test kit for SARS-CoV-2 has numerous advantages; for example, it saves time, is easy to carry out, additional equipment is not required, and it requires little training [72]. Further, in Figure 5B, a single line at the control (C) validates the authenticity of the test; thus, this line is a must during analysis, and G and M, respectively, represent IgG and IgM. Further, the single line at G and M represents previous and recent infection with SARS-CoV-2, respectively. In addition, lines at both G and M represent SARS-CoV-2 infection, and the absence of lines in both places represents no infection or early infection due to antibodies not being available in traceable amounts [72].

Qui, et al. reported a two-dimensional gold-nanoisland (AuNIs)-based nucleic acid sensor for detecting SARS-CoV-2. A dual-functional plasmonic biosensor was successfully fabricated and incorporated with localized surface plasmon resonance (LSPR) and a plasmonic photothermal (PPT) effect as transduction principles for sensing. This biosensor was fabricated using a 2D AuNIs chip following the self-assembly of thermally de-wetted Au nanofilm on the BK7 glass surface. The Au-film was firstly prepared via magnetron-sputtering. The LSPR and PPT can be excited at two different wavelengths through two different rent angles of incidence, which considerably amplifies the sensitivity, reliability, and sensing stability. Therefore, the suggested biosensor can provide an easy and reliable diagnosis platform; moreover, it alleviates the pressure of PCR-based tests. The proposed LSPR biosensor has superb sensitivity for detecting SARS-CoV-2 nucleic acid, with a detection limit of 0.22 pM [55]. Similarly, silver nanoparticles (AgNPs) are taken into consideration as one of the potential antiviral agents that can act against the lethal virus. AgNPs can be incorporated to the viral genome, obstructing the interaction and activity of numerous virus and cellular factors responsible for repetition and resulting in the prohibition of viral replication. By evaluating various properties such as shape, size, and surface charge, the interaction of AgNPs with viruses is enhanced [73]. AgNP-based biosensors could be employed in diagnostics and in the sensing of viral infection. Due to their catalytic, photonic, electronic, optical, and clinical applications, AgNPs are crucial in combating the SARS-CoV-2 virus [55,74]. Yang, et al. developed an ACE2-functionalized silver-nanotriangle (AgNT)-based LSPR sensor for detecting SARS-CoV-2 spike RBD with a detection limit of 0.83 pM. The fabricated biosensor consists of four steps: the fabrication of AgNT, the immobilization of ACE2 on AgNT, BSA blocking, and then, the detection of the virus or RBD protein. The developed sensor shows high specificity and sensitivity to CoV NL63 and SARS-CoV-2 [56]. Ghisetti, et al. reported on the POCT STANDARD Q COVID-19 Ag for rapid SARS-CoV-2 nucleoprotein detection in NP swabs, with the result appearing in less than 30 min; this was applied to 330 patients of two different populations from phase 1 and 3 of the pandemic. The main advantage of POCT for antigen testing is that it requires limited technical skill and infrastructure; furthermore, it is certainly preferable in point-of-care centers for mass screening of the SARS-CoV-2 virus. The study reported that 70.6% of the positive RT-PCR samples were not false-positive results, which were identified via STANDARD Q COVID-19 Ag [75]. Teengam, et al. reported AgNP paper-based colorimetric DNA sensors for detecting Middle East respiratory syndrome (MERS). MERS-CoV DNA was effortlessly determined using a cationic pyrrolidinyl peptide nucleic acid (acpcPNA) probe. The probe was positively charged with a lysine moiety present at the carboxyl terminus so it could interact with the negatively charged targeted DNA or AgNPs. In the case of DNA binding, it prevents aggregation without any color change, while in the case of AgNP binding, it leads to NP aggregation with a significant color change. This fabricated paper-based colorimetric DNA sensor could be another approach for easy, quick, sensitive, and selective DNA detection, and therefore, the fabricated sensor can serve as a POC diagnostic tool for viral DNA detection. In the same way, SARS-CoV-2 DNA may be detected by AgNPs [76,77]. One more work by Chen, et al. developed a simple and rapid lateral flow immunoassay based on lanthanide-doped nanoparticles (LNPs) to diagnose anti-SARS-CoV-2 IgG in serum samples within 10 min. This biosensing platform was fabricated through lanthanide-doped polystyrene NPs, developed using the mini-emulsion polymerization method. By using rabbit IgG (R-IgG) following a EDC/NHS chemical reaction and mouse anti-human IgG antibody (MH-IgG), the surface modification of LNPs was performed. LNPs have demonstrated several optical properties, as they possess remarkable electronic configurations along with high and sharp emission bands, which are extensively used for sensitive biosensing applications [57]. Likewise, various metal oxides were recently turned into significant materials for identifying several viral diseases, including influenza, ebola, and current COVID-19 [78,79]. Ishikawa, et al. reported that In_2_O_3_ nanowires were grown on Si/SiO_2_ substrate via the laser ablation CVD method to detect the SARS-CoV-2 N protein. The fabricated FET biosensor could detect N protein at subnanomolar concentrations, with a response time of ~10 min [80]. Similarly, transition metal oxide is extensively reviewed for its antiviral nature due to its stability, physiochemical properties, cost-effectiveness, and reasonable stability rate. Vadlamani, et al. developed a cobalt-functionalized TiO_2_-nanotube-(Co-TNTs)-based electrochemical sensor for detecting the SARS-CoV-2 spike-receptor-binding domain protein with a detection limit of 0.7 nM. The TNTs were fabricated via one-step electrochemical anodization of the Ti sheet and Co functionalization was performed using the wet ion-exchange method [8]. Neal, et al. synthesized two sliver-modified cerium oxide nanoparticles by using the base-mediated forced hydrolysis method for SARS-CoV-2, as the cerium oxide nanomaterial has demonstrated considerable biomedical efficiency [81]. The SARS-CoV-2 virus spreads through surfaces (such as handles, gas pumps, doors, and elevator buttons), and remains on the surface for up to 1 week; thus, surface transmission can be inhibited by using a coating. Coating the surface with cuprous oxide (Cu_2_O) inactivates 99.9% of SARS-CoV-2 [82]. For example, Hosseini, et al. fabricated cupric oxide (CuO), which diminishes infection from SARS-CoV-2. The porous film creates a good connection between the microbe and the CuO solid because of the highly sintered area. A thin coating of CuO, developed via the thermal oxidation of Cu_2_O, succeeded via sintering. The inactivity of the virus due to the coating of CuO was examined using Vero E6 cells [83]. It has become known that the SARS-CoV-2 virus is mainly transmitted from an infected person’s nose or mouth in small liquid particles when they cough, speak, sneeze, and breathe; these pass-through air and are small enough to remain airborne for hours. Therefore, there have been several reviews reported on breath analysis due to its potential for the screening, treatment, and diagnosis of SARS-CoV-2. Moreover, it could be used at home, in central facilities, and in POC, which lowers the burden on hospitals [84,85]. Thus, Haick, et al., in 2022, reported a AuNP-based sensor for screening and detecting the SARS-CoV-2 virus directly from exhaled breath. Recent studies have suggested that the microenvironments emitting volatile organic compounds were created by the viral agents of SARS-CoV-2. The various sensing layers developed by the link of AuNPs to organic ligands shrink and bulge upon exposure to VOCs; this causes changes in the electric resistance, which can be measured digitally. The proposed device needs frequent washing with alcohol and the sensor needs to be flushed with air after every use. The VOCs can target breath diagnostics without being invasive to patients. The study cohort included 14 symptomatic negative cases, 47 asymptomatic controls, and 41 confirmed cases of the SARS-CoV-2 virus [86].

### 3.2. Carbon-Based Nanobiosensor

Carbon-based nanomaterials, namely carbon nanotubes, graphene, and fullerene, are fascinating for antimicrobial application. They are favorable materials for combating SARS-CoV-2 viruses via various mechanisms because of their exceptional electrical conductivity, optical and biocompatible performance, etc. [87,88].

#### 3.2.1. Graphene

**Figure 6:**
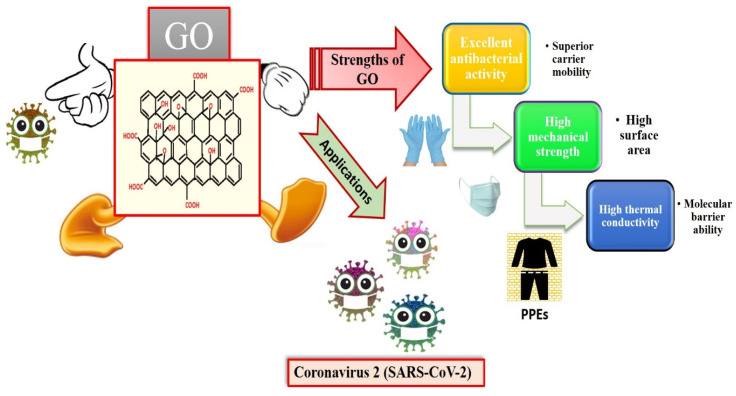
Applications of graphene oxides (GO) and their strengths (reproduced with permission from Hashmi, A., et al., 2022, Mater. Today Adv., 13, 100208 [51]).

**Figure 7:**
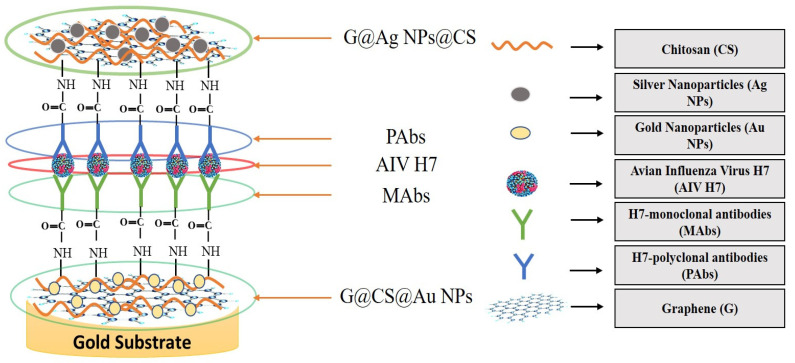
Illustration of sandwich-type immunosensor for determination of avian influenza virus H7 (reproduced with permission from Hashmi, A., et al., 2022, Mater. Today Adv., 13, 100208 [51]).

Graphene is an intriguing two-dimensional (2D) carbon material in which each carbon in graphene is sp2 hybridized, and the carbon atoms are covalently linked in a honeycomb lattice. Furthermore, it has attracted much consideration owing to its promising antiviral and antimicrobial applications; see Figure 6 for the strength of graphene oxides and their applications in combating SARS-CoV-2. However, the interactions among graphene derivatives and viruses are still disputable. Graphene materials are highly suitable for biosensing to detect viruses due to their unique characteristics and properties such as electrical and thermal conductivity, light weight, chemical stability, high optical transparency, their function as a zero-band-gap semiconductor, non-toxicity, and mechanical stability. Additionally, the large surface-area-to-volume ratio of graphene is advantageous for drug carrier applications, but it can also play a vital role in biosensing applications [89,90,91]. In biosensors, graphene-based nanomaterials are employed as transducers that convert the interactions among the target molecule and the receptor molecules into measurable signals [92]. For this to occur, molecules such as ssDNA, enzymes, and antibodies must be attached to the transducer surface. The most common approach for enzyme immobilization onto graphene, graphene oxide, and reduced graphene is physisorption, while ssDNA and antibodies are most commonly immobilized using EDC/NHS chemistry [93]. In several studies, graphene-based biosensing devices have already been reported to screen various infectious diseases, including SARS-CoV-2. Two-dimensional-material-based biosensors can be classified as optical-based devices, mainly relying on sensing via SPR, surface-enhanced Raman scattering (SERS), and fluorescence and electrode-based devices, including field-effect transistors (FET) and electrochemical biosensors [94]. Graphene-based FETs have mainly been significant due to their low noise detection, rapid estimation, even in the presence of small amounts of the analyte, and ultra-sensitivity [95]. For instance, by utilizing the properties of graphene, Seo, et al. fabricated a FET-based biosensor with a LOD of 1.6 × 10^1^ pfu/mL, and 2.24 × 10^2^ copies/mL in the case of SARS-CoV-2 spiked culture and clinical samples. The FET-based biosensing device was developed by coating graphene surface with a specific antibody of the SARS-CoV-2 spike protein. The fabricated graphene-based biosensor could detect the SARS-CoV-2 virus because the functionalized graphene-based biosensor provides rapid, highly responsive, and simple detection [1]. Huang, et al. [96] developed ultrasensitive (low limit of detection of 1.6 pg/mL) and selective graphene@chitosan@Au nanoparticles (G@CS@Au NPs) and G@Ag NPs@CS-based sandwich-type biosensors (immunosensor) for diagnosing avian influenza virus H7. In this sandwich-type immunosensor, H7-monoclonal antibodies (MAbs) are attached with G@CS@Au NPs, and H7-polyclonal antibodies (PAbs) are attached with G@Ag NPs@CS (Figure 7); this fabrication of a diagnostic approach for the detection of the virus led to the development of biosensors for virus determination. Further, Palmieri, et al. demonstrated that graphene could be one of the prime materials for combating the SARS-CoV-2 virus by interacting with the surface of the virus, its genetic material, and its proteins via diffusion of these materials to inhibit functions such as genome replication and protein synthesis, which damages the integrity of the virus [97]. Layqah, et al. developed an electrochemical immunosensor using a carbon electrode with gold nanoparticles to detect the MERS-CoV virus [98]. Yusof, et al. showed that graphene is an appropriate material to fight the spread of the SARS-CoV-2 virus, as biomass-derived graphene has enormous potential to be implemented as a sterilizer and can be used in face masks to inhibit any droplets from entering our body [99].

Similarly, Zhong, et al. reported graphene masks with ultra-hydrophobic and photothermal performance to fight the SARS-CoV-2 virus because the superhydrophobic surfaces provide enhanced protection against incoming respiration droplets [100]. Xu, et al. fabricated a multiplexed electrochemical graphene-based platform for the quick and selective identification of the spike protein (S1) and nucleocapsid protein (NP) of the SARS-CoV-2 virus. The multiplexed platform exhibits great potential to perform at point-of-care and permits an at-home assay for COVID-19 detection for telemedicine administration and control [101]. Zhao, et al. used calixarene-functionalized graphene oxide to fabricate an ultrasensitive electrochemical biosensor for detecting the RNA of SARS-CoV-2, which has promising applications in point-of-care diagnostics. The study reported 88 RNA extracts from 25 SARS-CoV-2-confirmed patients [58]. Payandehpeyman, et al. reported a nanoresonator sensor for detecting the spike S1 antigen of SARS-CoV-2 based on graphene. The anti-SARS-CoV-2 Spike S1 antibody was immobilized on a single-layer graphene sheet (SLGS) surface that functioned as a sensing area. Additionally, a nanoresonator based on graphene offers highly sensitive and quick identification of SARS-CoV-2 [59]. Kim, et al. fabricated a graphene-enabled biosensor for quick and simple determination of the SARS-CoV-2 virus using a quasi-freestanding bilayer epitaxial graphene and optimized immobilization circumstances of the heterostructure spike S1 protein antigen of SARS-CoV-2 [102]. A group of researchers has developed and reported a graphene-based FET device for identifying the spike protein of SARS-CoV-2, as graphene-based FET devices are quick, highly responsive, and easy to operate for the determination of the SARS-CoV-2 virus in clinical samples. The FET-based biosensing platform was developed using a graphene channel conjugated to the spike protein S1 antigen of SARS-CoV-2, and FET was dipped in phosphate-buffer saline. Moreover, the FET sensor determined the spike protein of SARS-CoV-2 with a detection limit (LOD) of 1fg/ML [1,103]. Graphene not only has an essential role in dealing with the current virus, but also provides a technical instrument for detecting, controlling, and protecting against SARS-CoV-2 and forthcoming viral infections.

#### 3.2.2. Carbon Nanotubes

Carbon nanotubes (CNTs) can be described as allotropes of carbon that comprise a hollow, cylindrical structure of a hexagonal network of carbon atoms. The structure of CNTs belongs to the fullerene family, and carbon atoms in nanotubes are sp^2^ hybridized. Moreover, carbon nanotubes can be categorized into multi-walled (MWCNTs) and single-walled carbon nanotubes (SWCNTs) [104,105]. Carbon nanotubes exhibit an array of applications in biosensors, drug delivery, and diagnostics with greater longevity because of their cheaper production and biological, magnetic, electronic, mechanical, and optical properties, compared to the bulk material [106,107]. The unique properties of CNTs have stimulated the development of CNT-based biosensors. CNTs can be employed as transducer and electrode materials in biosensors due to their electrochemical and electrical properties [108]. The CNT-based biosensor comprises two parts: a biologically sensitive element and a transducer. The CNT functionalized with a bioreceptor or biomolecules (for example, proteins, microorganisms, or oligo- or polynucleotides) acts as the biologically sensitive element, and the transducer converts the concentration of analytes to detectable signals [109]. The application of CNTs in the biosensor field is focused on field-effect, optical, and electrochemical devices [110]. Zamzami, et al. fabricated a CNT-based field-effect transistor (FET) electrochemical biosensor to acquire the spike S1 antigen of SARS-CoV-2 in buffer solution. The biosensor was fabricated on a Si/SiO_2_ surface using CNT printing, and the SARS-CoV-2 S1 antibody was immobilized on the CNT surface between the S and D sensing areas. The LOD of the fabricated CNT-FET biosensor was 4.12 fg/mL. CNT-FET-based biosensors are reliable, easy to fabricate, cheap, and can quickly detect SARS-CoV-2. Furthermore, CNT-FET-based biosensors are an extraordinary device for detecting ongoing infection [60,111]. Likewise, Thanihaichelvan, et al. reported a CNT-FET-based biosensor for the selective determination of SARS-CoV-2 RNA, while CNT-FET was fabricated on a Kapton substrate [112]. Shao, et al. demonstrated that semiconducting-SWCNT-based FET could detect the spike protein of SARS-CoV-2 in less than 5 min. Moreover, the reported semiconducting-SWCNT-FET provided higher analytical sensitivity toward the target analyte [113]. Another work was reported by L. Pinals, et al. using a near-infrared mechanism to develop an angiotensin-converting enzyme (ACE2) SWCNT-based optical nanosensor, which was highly bound to the spike protein of SARS-CoV-2 [53,114]. Aasi, et al. reported metal-decorated SWCNTs for the adsorption of hydrogen peroxide (H_2_O_2_) as an effective platform to combat viruses. The stability of the sensor was improved by incorporating metallic nanoparticles into the CNT-based sensor [115]. Abdolahad, et al. fabricated an MWCNT-based electrochemical sensor to detect the intensity of mitochondrial reactive oxygen species (ROS) in a sputum sample with a volume of less than 500 µL. An integrated sensor whose working electrode was coated with functionalized MWCNTS was entered into a sputum sample and the ROS peaks were recorded. The study reported that more than 97% of positive patients were detected, and it took less than 30 s. Moreover, it has great potential for rapid screening of the SARS-CoV-2 virus [116]. Thus, the CNT-based sensors can be an efficient tool to diagnose the SARS-CoV-2 virus via optical or electrochemical sensing mechanisms, and researchers working on these aspects can focus on developing a more reliable, selective, and sensitive nanobiosensing device for combating SARS-CoV-2 infection [117,118].

#### 3.2.3. Fullerene

A fullerene is an allotropic form of a carbon atom shaped like a sphere, ellipsoid, and other shapes. The most abundant form of fullerene is buckminsterfullerene (C60), with 60 carbon atoms arranged in a spherical structure, which has exhibited promising binding affinity toward the SARS-CoV-2 [87,119]. Fullerene has drawn significant attention and excitement in biomedical and medicinal chemistry, and in drug delivery. It also shows antiviral performance due to its low side effects, excellent biological performance, and antioxidant capability. Hurmach, et al. reported the potential binding mechanism between the RdRp and 3CLpro proteins of SARS-CoV-2 with C60 fullerene. The simulation showed that C60 fullerenes can hinder the RdRp and 3CLpro protein of the SARS-CoV-2 virus using a distinct mechanism [120]. Further, Bagheri Novir, et al. reported the interaction between the chloroquine drug and pristine fullerene, and they also explored the interaction between the chloroquine drug and aluminum-, silicon-, and boron-doped fullerene using DFT calculations. For chloroquine, aluminum- and silicon-doped fullerenes appeared to be good delivery systems in the treatment of SARS-CoV-2 [121]. Fullerenes are not much explored for developing nanobiosensing systems because of a few challenges, such as the complex functionalization of fullerene, the cost of fullerene, the stability of the sensor morphology under different conditions, and the multidetermination or multiplexing of more than one analyte [122]. However, if these materials are utilized for developing nanobiosensing devices for detecting SARS-CoV-2, they could prove to be promising for determining SARS-CoV-2 with high selectivity and sensitivity [123]. Thus, researchers working on the development of biosensing approaches can work toward this aspect in relation to fullerene-based materials.

### 3.3. Quantum-Dot-Based Nanobiosensors

**Figure 8:**
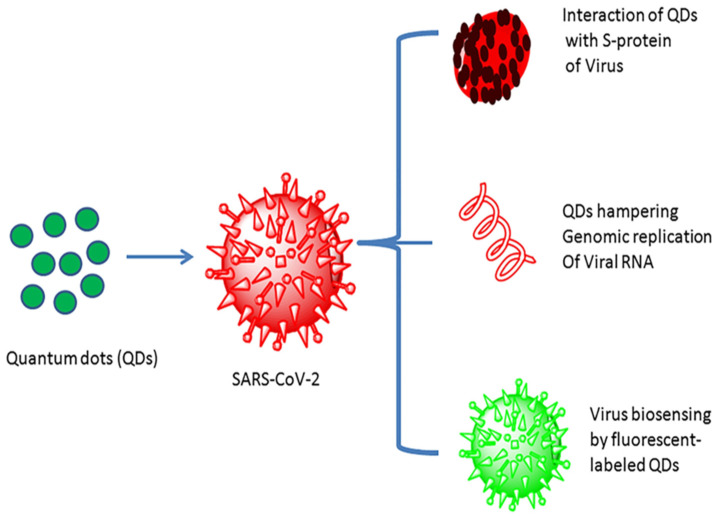
Quantum dot interaction with the SARS-CoV-2 virus and their utility (reproduced with permission from Manivannan, S. and Ponnuchamy, K., 2020, Appl. Organomet. Chem., 34 [127]).

Quantum dots (QDs) were first ascertained around the beginning of the 1980s. Quantum dots are inorganic semiconductor materials conjugated with high fluorescent probes, usually in the range of 1 to 10 nm with a tunable optical wavelength. Furthermore, quantum dots are often cited as artificial atoms [124]. Quantum dots have captured enormous attention in nanotechnology, biotechnology, and bioimaging applications, with high sensitivity, selectivity, and rapidity because of their unique optical and electronic properties [125,126]. Quantum dots have been considered promising for sensing and treating SARS-CoV-2 and other viral infections, as their low toxicity and high surface performance make them supreme amongst other nanomaterials (Figure 8) [127]. Quantum-dot-based nanomaterials, for example, fluorescent, graphene, and carbon quantum dots, have also attracted considerable attention for developing an efficacious biosensor for detecting various viral infections due to their unique properties [128].

Boroushaki, et al. fabricated a carbon electrochemical sensor to fabricate a screen-printed electrode reinforced by boron nitride quantum dots/a flower-like gold nanostructure. The developed electrochemical biosensor has the potential for the sensitive and accurate diagnosis of SARS-CoV-2 in laboratory samples [61]. Further, Zheng, et al. developed a fluorescent quantum dot coupled with the B-cell epitopes of SARS-CoV-2 to determine SARS-CoV-2 with a detection limit of 100 pM. B-cell-epitope-based quantum dot biosensors can vigorously separate coronavirus diseases compared to the conventional ELISA. Fluorescent quantum dots have the potential to detect antigens and antibodies quickly [129]. Graphene quantum dots (GQDs) are nanoscale structures of graphene with a size of less than 100 nm, which show outstanding properties such as exceptional biocompatibility; electronic, optical, and quantum confinement; low toxicity; high water solubility; and multicolored emission [130,131]. Owing to the aforementioned properties, graphene quantum dots have potential in biomolecule detection, drug delivery, and the biological imaging of numerous viral infections, and are promising for nanotechnological development [132]. Graphene quantum dots can be synthesized either via bottom-up or top-down methods [133]. Song, et al. synthesized N-doped graphene quantum dots (NGQDs) via a microwave-assisted hydrothermal reaction for the transfection of several genes, as the graphene quantum dots provide a promising platform for drug delivery [134]. Ray, et al. synthesized human cathelicidin LL-37 and neutrophil α-defensin HNP1 (a human host defense peptide) attached to GQDs. GQDs inhibit the delta variant’s virus by accessing host cells and prohibiting the spike protein RBD from binding with ACE2 due to the GQDs’ unique properties [135]. Li., et al. fabricated a magnetic relaxation switch (MRSw) to detect and eradicate the antigen S protein of SARS-CoV-2 based on GQDs. This approach gives quick, safe, and extremely sensitive identification of the SARS-CoV-2 virus [136]. Carbon quantum dots (CQDs), also called carbon dots, are carbon-based nanomaterials that are small nanoparticles with an average size below 10 nm. CQDs have shown tremendous potential in various applications such as photothermal therapy, drug delivery, bioimaging, and biosensing, due to their state-of-the-art properties for example, conductivity, optical detection, high chemical stability, outstanding biocompatibility, and low cost. Furthermore, with brilliant accuracy, they have demonstrated the incredible capability to detect and exterminate several pathogenic viruses, including the SARS-CoV-2 [137]. Loczechin, et al. developed carbon-based quantum dots for the potential diagnosis of human coronavirus (HCoV-229E), and boronic acid improved CQDs [138]. Thus, QDs can play a significant role in determining SARS-CoV-2, but are not explored fully in terms of SARS-CoV-2 diagnosis [139,140]. Hence, researchers focusing on the applications of various QDs, such as CQDs and GQDs, can explore their utility for determining contagious viral infections such as SARS-CoV-2 and MERS-CoV.

### 3.4. Polymer-Based Nanobiosensor

Polymer-based nanomaterials are developed from a polymeric material with the help of colloidal organic compounds at the nanoscale and are synthesized in a nanosphere or nanocapsule structure [141]. Recent research attempts have stated the substantial performance of polymer-based nanobiosensors for detecting viruses and the development of vaccines and drugs due to their excellent biocompatibility, biomimetic applications, exclusive physical and chemical properties, non-toxicity, biodegradability, and ultra-sensitivity [142,143]. Additionally, polymer-based nanomaterials are highly decomposable, protecting the antigens or drugs from degradation. The analyte-recognition elements in biosensors are generally built on biological macromolecules such as antibodies, aptamers, enzymes, and DNA. Nevertheless, such bioanalytical techniques possess certain shortcomings due to their high price of production [144,145]. A variety of approaches has been thoroughly examined to substitute these biological receptors with synthetic analogs. Therefore, artificial biorecognition systems based on molecularly imprinted polymers (MIPs) have been established, and have attracted attention as a probable substitute in recent years. Molecularly imprinted polymers are developed using the molecular imprinting method, which can be described as a method of template establishment of particular molecular recognition sites in a polymer material [146]. MIP is a technique for manufacturing affinity polymers for different targets of analytical interest. It includes the development of a complex among a given target molecule and functional monomers through covalent, non-covalent, or semi-covalent interactions, which are then subjected to polymerization. After polymerization, the target molecule is eliminated from the imprinted material, leaving specific cavities corresponding to the template in shape, chemical functionality, and size [147]. Furthermore, molecular imprinting technology enables the preparation of MIP-based artificial antibodies capable of identifying and binding various microorganisms comprising viruses and microbes [148]. MIPs have been employed in many applications, such as isolation, purification, biosensors, and catalysis [149,150]. The MIP-based sensors fabricated in the presence of the target molecule are effective in detecting target molecules through their specific cavities and are consistent with the target molecule. There are several research reports on MIP-based biosensors, which have the great potential to detect various types of viruses, namely the human immunodeficiency, influenza, dengue, and hepatitis viruses [151]. For instance, Tancharoen, et al. fabricated a Zika virus (ZIKV) electrochemical sensor based on an imprinted graphene oxide polymer. A fabricated biosensor was employed to detect ZIKV by measuring the electrical signal with varying virus concentrations in serum and buffer, employing a standard electrochemical method [152]. Ma, et al. fabricated an MIP electrochemical sensor constructed on the surface of an MWCNT for detecting HIV-p24 in human serum samples, with an LOD of 0.083 pg cm^−3^. This sensor was successfully utilized to detect HIV-p24 and possessed good selectivity, reproducibility, and repeatability [153]. This identical method can be employed to determine the definite aptamers for SARS-CoV-2 due to the sudden changes that are currently being observed. At the moment, some exploration reports are already applying MIP technology to SARS-CoV-2 determination. High selectivity, robustness, prolonged stability, cost-efficiency, and an affinity for the targeted molecules are the most significant advantages of MIPs [154,155]. Parisi, et al. developed nanoparticle-based MIPs that can selectively bind to a part of the spike protein of SARS-CoV-2. In addition, the prepared nanoparticles could be used for treating and inhibiting SARS-CoV-2 without any use of drug. It also has a noteworthy capability to exert antiviral activity in vitro [156]. Sharif, et al. developed electropolymerized MIPs (E-MIPs) to selectively detect SARS-CoV-2 using acrylamide-based hydrogel as a functional monomer with an LOD of 4.9 log_10_ pfu/mL. Via reductive electropolymerization, the E-MIPs, imprinted with SARS-CoV-2 pseudoparticles (pps), were electrochemically deposited onto screen-printed electrodes, crosslinked with N,N′-methylenebisacrylamide (MBAm) and water-soluble N-hydroxmethylacrylamide (NHMA) as a functional monomer. The developed E-MIPs capture the virus from patient saliva samples [157]. Ramanavicius, et al. fabricated a molecularly imprinted polymer-based (polypyrrole) sensor to detect the SARS-CoV-2 spike glycoprotein [148]. Raziq, et al. fabricated an electrochemical sensor based on a molecularly imprinted polymer (MIPs) for examining the nasopharyngeal swab specimens of patients with a LOD of 15 fM. The developed sensor represents a portable diagnostic platform for the quick screening of SARS-CoV-2 ncovNP (nucleoprotein). The sensor was fabricated in a cell phone or tablet for monitoring [62]. Likewise, Syritski, et al. fabricated an electrochemical sensor with detection limits of 15 fM and 64 fM to identify the spike protein S1 (ncovS1) of SARS-CoV-2 [63].

Furthermore, an MIP-based electrochemical sensors have excellent potential for on-the-spot analysis of SARS-CoV-2 patients; therefore, researchers must pay attention to the development and optimization of MIP synthesis parameters to make them more selective and sensitive toward SARS-CoV-2 viral antigens so that they can be more efficiently utilized for developing electrochemical nanobiosensing devices [63].

### 3.5. G-Quadruplex-Based Nanobiosensors

G-quadruplex is a non-canonical nucleic acid structure fabricated by coupling four guanine bases through the Hoogsteen hydrogen bond. In recent years, G-quadruplex has been significantly employed in biosensors for detecting nucleic acids, biological enzymes, metal ions, and organic molecules due to their low cost, simple operation, low detection limit, high sensitivity, and fast detection ability [158,159]. G-quadruplex has been shown to detect various viruses, such as HIV, hepatitis A (HAV), and SARS-CoV, because the G-quadruplex structure has been shown to influence the entire life cycle of the virus, including recombination, gene-expression control, and replication. Therefore, G-quadruplex is also a promising potential tool for SARS-CoV-2 detection [160,161]. G-quadruplex can be considered a probe in biosensors that identifies proteins, nucleic acids, and metal ions by changing binding events into detectable signals. A G-quadruplex-based biosensor comprises a recognition element and converter whereby the recognition element is the G-quadruplex nucleic acid applied to distinguish the target for the examination and converter, which can transform the biological binding event into the vibrational, optical, and electrical signals for observation [162,163]. G-quadruplex-based biosensors include optical and electrochemical biosensors. G-quadruplex-based biosensors have easy regeneration, low cost, sensitivity, and stability, and high affinity compared to other common biosensors [164]. G-quadruplex structures seem to be a promising substitute method for SARS-CoV-2 detection based on the aptamer probe ligation test; moreover, this method does not involve the nucleic acid extraction step. Currently, 25 putative G-quadruplex-forming sequences (PQSs) in the genome of SARS-CoV-2 are recognized, which are positioned in the open reading frames of ORF3a, ORF1ab, N, S, and M genes of SARS-CoV-2 [165,166,167]. The detected top-ranked PQSs were shown to form RNA G-quadruplex structures in vitro via multiple spectroscopic assays. Moreover, the direct interaction of the SARS-CoV-2 G-quadruplex structures with a viral helicase was shown via microscale thermophoresis. In addition, the G-quadruplex sequence identified in SARS-CoV-2 could also be utilized in the biosensors for identifying the viral helicase protein nsp13 of SARS-CoV-2 [168,169].

## 4. Challenges and Limitations

Regardless of biosensors’ triumph in screening and spotting a broad array of viruses, certain limitations and challenges still prevail, which restrain the sensing of various viruses. Biological samples generally comprise molecules such as nucleic acid and protein, but currently, most analyses merely perceive one analyte at a time. Therefore, attention needs to be given to enhancing efficacy, sensitivity, and selectivity; moreover, it is important to concurrently examine numerous analytes because various assays can acquire more information [170]. Traditional tools for respiratory virus detection are the most precise, extremely sensitive, and quantitative assay for SARS-CoV-2 RNA, and are accessible solutions for the earliest assay. Nevertheless, they are highly priced, time-consuming, require skilled laboratory personnel and intense work, are not appropriate for point-of-care diagnostics, and may cause a false-negative result [171,172]. For example, RT-PCR was working as the primary analytic tool for the SARS-CoV-2 pandemic and acceptable analytical results were received, but this method had many restrictions such as a lack of reliability; moreover, the assembly of all the spiked samples is not patterned. Hence, RT-LAMP is implemented as an alternative to RT-PCR; it provides rapid diagnosis and a colorimetric assay, but the method relies on different primers and is not beneficial for mass testing [173]. One of the most significant difficulties in fabricating appropriate biosensors is apprehending a small magnitude signal within the analyte and bio-receptor. Nanomaterials can be exploited in biosensors because of their unique properties (such as electrical, optical, physical, and chemical properties) to enhance the selectivity of the biosensor. For example, metallic nanomaterials (e.g., gold, copper, and silver), carbon-based nanomaterials, quantum-dot-based nanomaterials, and polymer-based nanomaterials may be utilized for identification by attaching them to the targeted DNA probe. So, the nanomaterial-based biosensor can be implemented in place of a PCR-based assay for SARS-CoV-2. Furthermore, developing biosensors based on biological reagents is a time-consuming and costly procedure [174,175]. Annually, several nanomaterial-based biosensors are reported, but just a few of them are efficiently used in laboratory assays. The significant challenges of the nanomaterial-based biosensor are reproducibility and an extremely low detection limit. Additionally, the synthesis of nanomaterials is complex and proficiency-demanding, and yields are scarce and expensive [176]. Because of the high price of raw materials such as CNTs, some of the nanobiosensors are not cost-efficient, which restrains the fabrication of nanobiosensor to commercialization. Moreover, the sensitivity of nanobiosensors to various fabrication methods and their size lead to a technical challenge in producing nanobiosensors. Nanobiosensors based on biological reagents require an expensive process of extraction and/or preparation of target biological reagents [177]. Fundamental knowledge of the influence of nanobiosensors in a biological setting is inadequate. Therefore, before developing any nanobiosensors for large-scale applications, it is necessary to study their biological relevance. There is a poor understanding of the cytotoxic and detrimental impact affiliated with nanobiosensors [178]. Furthermore, it is hard to achieve high-quality and purified graphene at an industrial scale to produce a significant number of graphene-based biosensors. For higher sensitivity of these devices, the purity of graphene is a considerable challenge, and any error in the fabrication process can lead to inaccurate biosensors. Electrochemical-based biosensors could not detect an analyte of low concentration, and the matrix may impact the outcome in an inaccurate measurement [179,180,181]. In addition, electrochemical-based biosensing includes sample damage during analysis, high cost of the instrument with low detection limits, and the time-consuming preparation of electrodes [182]. Further investigation for laboratory and commercial applications of QDs is required; mainly, their safety aspects have not been represented appropriately to perceive their interactions with other molecules (such as nucleic acids). To augment the use of QD-based biosensors in the treatment and detection of SARS-CoV-2, many challenges have yet to be resolved, for instance, the large-scale production of nanomaterials with high reproducibility, long-term storage stability, and sensitivity [183]. Nevertheless, LSPR is a sensitive and adaptable method, but some issues still prevent its use. The first major challenge in LSPR biosensing is the detection of small molecules, which need to be present in considerable numbers to coat the surface, and limited sensitivity and detection limits, continue to be problematic. Another key challenge is the selectivity in binding the analyte of interest and integrating LSPR devices into multiplexed platforms [184,185]. Thus, more efforts are needed to better understand the estimation of nanomaterial-based biosensors for the development of diagnostic methods, which is important for controlling the ongoing pandemic. The research community is still striving to attain the ideal test because each of these tests has shortcomings.

## 5. Conclusions and Prospects

The COVID-19 pandemic has attracted scientists’ attention to, prompting them to make efforts towards the advancement of a better technique that must be sensitive, highly effective, and should be able to address the ongoing requirement of early diagnosis. Currently, no medicine or drugs are accessible to cure the ongoing virus. The best way to deal with this epidemic is via prompt screening, inhibition, and detection. Therefore, researchers have made every effort to develop and design effective, selective, sensitive, low-priced, extremely reliable, and correct methods to make a prompt analysis and to differentiate between infected and non-infected cases of COVID-19. In this review, traditional techniques are substituted with various sensing techniques comprised of nanomaterial-based biosensors for the detection of SARS-CoV-2 due to its limitations. Since nanomaterial-enabled biosensors have previously proven their ability to identify different diseases, they subsequently accomplished the ongoing requirement for the initial diagnosis of SARS-CoV-2. Several nanomaterials, such as polymer-based, quantum-dot-based, carbon-based, and metal-based nanomaterials, may be effectively applied in biosensing methods to analyze the SARS-CoV-2 virus. Regardless of these exclusive advantages, nanomaterial-based sensors for detecting COVID-19 have few shortcomings. To enhance the accuracy of SARS-CoV-2 detection, further efforts must be made. Hence, this review has focused on the application and function of several nanomaterial-based biosensing methods for determining SARS-CoV-2, along with its challenges and prospects.

## Figures and Tables

**Table 1:** Characteristics of nanobiosensors for detection of SARS-CoV-2 virus.

S. No.	Type of Sensor	Core Nanomaterials	Biomarker	Limit of Detection	Detection Time	Ref.
1	Plasmonic-effect-based colorimetric biosensing	Gold NPs	Thiol-modified antisense oligonucleotides (ASOs) specific for nucleocapsid phosphoprotein (N-gene) of SARS-CoV-2	0.18 ng/µL	10 min	[54]
2	Combined effect of plasmonic photothermal (PPT) and localized-surface-plasmon resonance (LSPR) biosensing	Gold NPs	SARS-CoV-2 nucleic acid/thiol-cDNA	0.22	15–20 min	[55]
3	LSPR biosensing	Silver NPs	SARS-CoV-2 spike RBD protein	0.83 pM	20 min	[56]
4	Lateral flow immunoassay based on fluorescence biosensing	Lanthanide-doped polystyrene NPs	Protein	-	10 min	[57]
5	Electrochemical biosensing	Cobalt functionalized TiO_2_	SARS-CoV-2 spike RBD protein	~0.7 nM	30 s	[8]
6	Electrochemical biosensing	Graphene oxide	RNA of SARS-CoV-2	200 copies/mL	-	[58]
7	Nanoresonator biosensing	Graphene	SARS-CoV-2 spike S1 antigen	10 copies per test	-	[59]
8	Field-effect transistor-based amperometric biosensing	Graphene	SARS-CoV-2 spike protein S1	1.6 × 10 ^1^ pfu/mL in spike culture 2.42 × 10 ^2^ copies/mL in clinical samples	5–8 min	[1]
9	Electrochemical biosensing	Carbon nanotube	SARS-CoV-2 spike S1 antigen	4.12 fg/mL	2–3 min	[60]
10	Electrochemical biosensing	Quantum dots	RNA of SARS-CoV-2	-	-	[61]
11	Electrochemical biosensing	Polymer nanomaterials	SARS-CoV-2 nucleoprotein	15 fM	-	[62]
12	Electrochemical biosensing	Polymer nanomaterials	SARS-CoV-2 spike protein subunit S1	15 fM in buffered saline and 64 fM in nasopharyngeal	-	[63]
13	Circle-to-circle-amplification-based optomagnetic biosensing	Iron oxide NPs	DNA of SARS-CoV-2	0.4 fM	~100 min	[64]
14	LSPR biosensing	Gold NPs	SARS-CoV-2 nucleocapsid (N) protein	150 ng/mL	5 min	[65]
